# The andean anticancer herbal product BIRM causes destabilization of androgen receptor and induces caspase-8 mediated-apoptosis in prostate cancer

**DOI:** 10.18632/oncotarget.12393

**Published:** 2016-10-01

**Authors:** Nagarajarao Shamaladevi, Shinako Araki, Dominic A. Lyn, Rajnikanth Ayyathurai, Jie Gao, Vinata B. Lokeshwar, Hugo Navarrete, Bal L. Lokeshwar

**Affiliations:** ^1^ Departments of Urology and Sylvester Cancer Center, Miller School of Medicine, University of Miami, Miami FL, USA; ^2^ Northwest Georgia Physicians Group, Gainesville, GA, USA; ^3^ Georgia Cancer Center and Department of Medicine, Augusta University, Augusta GA, USA; ^4^ Department of Biochemistry and Molecular Biology, Medical College of Georgia, Augusta University, Augusta GA, USA; ^5^ Herbarium QCA, Pontificia Universidad Catolica del-Ecuador, Quito, Ecuador; ^6^ Dr. Shinako Araki is at Okayama University graduate school of Medicine, Okayama, Japan

**Keywords:** Anti-cancer herbal preparation, androgen receptor, prostate cancer, chemoprevention, caspase-8

## Abstract

BIRM is an anticancer herbal formulation from Ecuador. Previous study established its antitumor and antimetastatic activity against prostate cancer models. The activity of BIRM against human prostate cancer (PCa) cells was investigated to uncover its mechanism of antitumor activity. In androgen receptor (AR)-expressing PCa cells BIRM was 2.5-fold (250%) more cytotoxic in presence of androgen (DHT) compared to cells grown in the absence of DHT. In AR-positive cells (LAPC-4 and LNCaP) BIRM caused a dose and time-dependent down-regulation of AR and increased apoptosis. Exposing cells to BIRM did not affect the synthesis of AR and AR promoter activity but increased degradation of AR via proteasome-pathway. BIRM caused destabilization of HSP90-AR association in LAPC-4 cells. It induced apoptosis in PCa cells by activation of caspase-8 via death receptor and FADD-mediated pathways. A synthetic inhibitor of Caspase-8 cleavage (IETD-CHO) aborted BIRM–induced apoptosis. The effect of BIRM on AKT-mediated survival pathway in both AR+ and AR- negative (PC-3 and DU145) showed decreased levels of p-AKT^ser 473^ in all PCa cell lines. BIRM dosed by oral gavage in mice bearing PC-3ML tumors showed selective efficacy on tumor growth; before tumors are established but limited efficacy when treated on existing tumors. Moreover, BIRM inhibited the LNCaP tumor generated by orthotropic implantation into dorsal prostate of nude mice. Partial purification of BIRM by liquid-liquid extraction and further fractionation by HPLC showed 4-fold increased specific activity on PCa cells. These results demonstrate a mechanistic basis of anti-tumor activity of the herbal extract BIRM.

## INTRODUCTION

Systematic investigations of promising plant products have led to the discovery and development of several anti-cancer agents with unique modes of action (e.g., paclitaxel, vinblastine, camptothesin, etc.) [1]. Prostate cancer strikes the elderly men (median age > 69) many of whom already have co-morbid conditions such as diabetes and heart disease, which make them less tolerant to aggressive treatments [2–4] including chemotherapy, radiation and even hormonal therapies. It is estimated that both in Europe and the United States, over 50% of the men with PCa experiment with one or more complementary therapies. These treatments include high dose vitamins and minerals, herbal preparations and supplement of soy, saw palmetto, and other supplements of less defined efficacy [5–8]. In addition, some of the recent products which have been shown to slow the development and progression of cancers of the prostate, breast, colon and lung include lycopene from tomatoes, curcumin from turmeric roots, phenethyl isothiocyanate and indole-3-carbinol from cruciferous vegetables [9–17]. Interestingly, many of these products are active ingredients in dietary supplements and herbal formulations in indigenous medicines. However, recommending the efficacy of complementary treatments without preclinical evaluation is not only inexact but also dangerous.

BIRM is an aqueous extract of dried roots of an Ecuadorian medicinal plant, *Kalanchoi gastonis-bonnieri.* BIRM is extensively used in Western Hemisphere mainly as Herbal immune booster, as by the manufacturer (Ecua-BIRM Inc., Quito, EC and on their website [WWW.EcuaBIRM.Com]. In addition, as per the manufacturer of BIRM, this product has been dispensed as a complementary medicine for ailments such as AIDS, lupus, arthritis related psoriasis and various treatment-refractory cancers. However, the mechanism of BIRM action and whether it is a cancer chemopreventative is unknown. We previously reported anti-tumor activity of BIRM against prostate cancer models, and have shown that it inhibits proliferation of all PCa cells, induces apoptosis and inhibits extracellular matrix (ECM) degrading enzyme, hyaluronidase [18]. Further, we showed that BIRM inhibits tumor growth and metastasis of Dunning MAT LyLu rat prostate tumor with no observable adverse effect. The mechanism of BIRM inhibitory activity against PCa, both *in vitro* and *in vivo*, has opened a new avenue for us to investigate the mechanism of anti-tumor activities of BIRM.

In this report we present evidence that BIRM inhibits growth of human PCa in subcutaneous and orthotopic models by oral administration. We further provide evidence that it inhibits AR mediated growth signaling by destabilizing AR-HSP-90 association and accelerated AR degradation. In addition, we demonstrate the mechanism by which BIRM induces apoptosis by the extrinsic cell death-induction pathway.

## RESULTS

### BIRM is more cytotoxic in the presence of androgen

**Figure 1:**
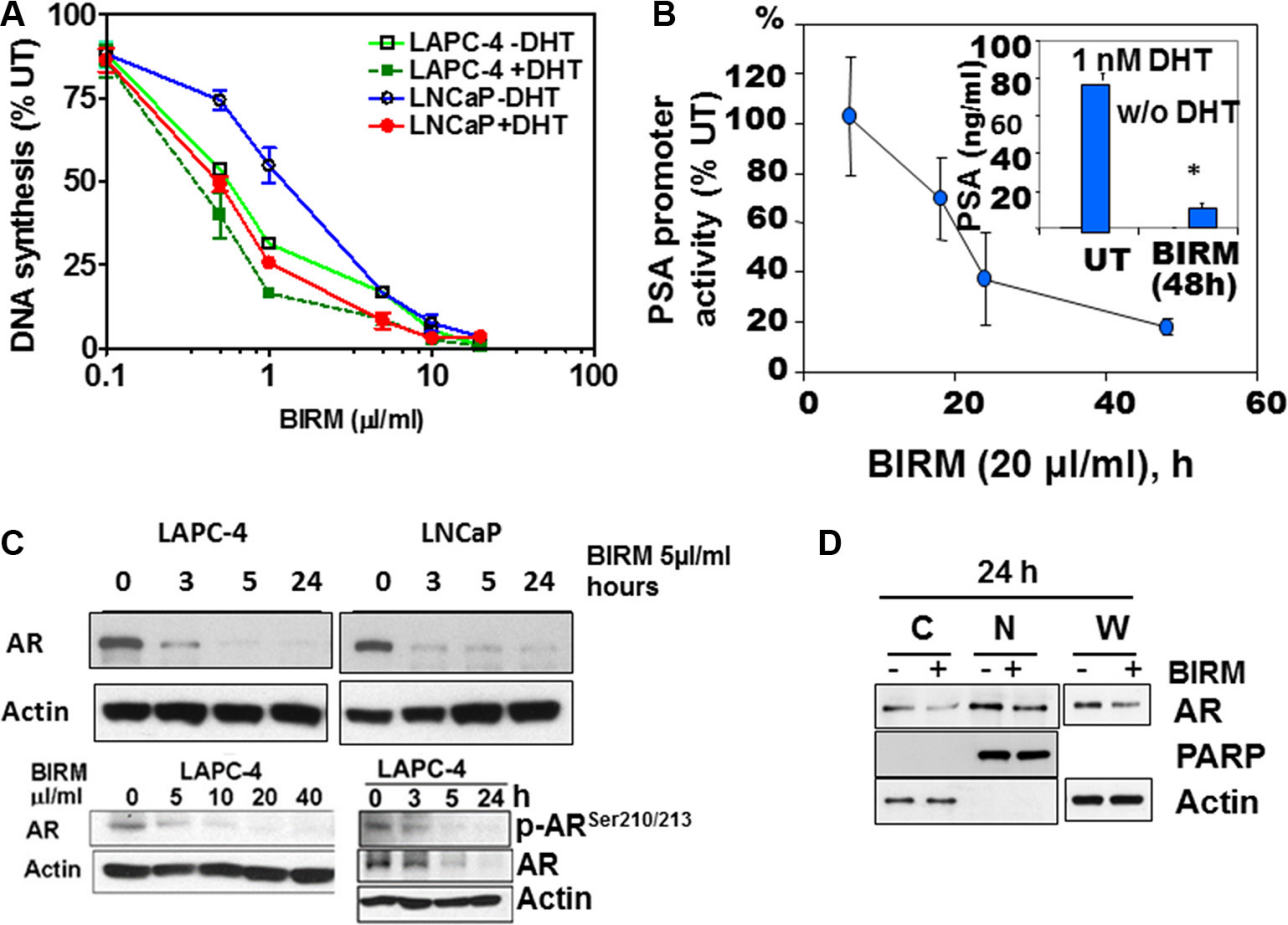
BIRM mediated AR down regulation (**A**) DNA synthesis inhibition in LAPC-4 and LNCaP cells treated with indicated dose of BIRM for 48 h with or without DHT were measured by [^3^H] Thymidine incorporation assay. (**B**) LAPC-4 cells were transiently transfected with PSA-luc and TK-Renilla. 24 h later, cells were exposed to 10 μl/ml BIRM in medium with 1 nM DHT for indicated time points and promoter activity was monitored by Dual-luciferase assay. Luciferase activity was normalized by Renilla activity and% promoter activity was measured compared to untreated (left panel). LAPC-4 cells were treated with 10 μl/ml BIRM in medium with or without DHT for 48 h and PSA production was monitored by ELISA (Inset). *p = 0.002; (**C**) Androgen dependent LAPC-4 and LNCaP cells cultured in growth medium were exposed to 10 μl/ml BIRM for indicated time point (left panel) or treated with various concentrations of BIRM for 24 h. Cells were harvested after incubation time and AR levels were analyzed by Immunoblotting. Actin was used as loading control. (**D**) LAPC-4 cells were treated with 10 μl/ml BIRM for 24 h and cytosol and nuclear fractions were prepared for immunoblotting to examine the effect of BIRM on AR localization. C: cytoplasmic AR, N: Nuclear AR in (A) and (C), the data presented as means ± SEM from two or more independent experiments in triplicate.

Previously, we reported dose-dependent inhibition of proliferation by BIRM in established PCa cell lines. The 50% growth inhibition determined by DNA synthesis activity (IC_50_) was 3 μl/ml (0.3% v/v) for LNCaP, and 8 μl/ml (0.8% v/v) for DU145 and PC-3ML cells [18]. This observation prompted us to inquire whether cytotoxicity of BIRM is dependent on the AR-mediated growth signaling in androgen responsive LNCaP and LAPC-4 cells. As shown in Figure 1A, BIRM at 1 μl/ml caused 45% and 69% decrease in DNA synthesis activity in LNCaP and LAPC-4 respectively, in the absence of DHT whereas 75% and 85% inhibition was observed in cells cultured with DHT, respectively. Therefore, we tested whether BIRM-induced growth inhibition is mediated through DHT-dependent AR growth signaling in androgen responsive PCa cells.

Indeed, as shown in Figure 1B, BIRM significantly inhibited the PSA promoter activity by 50% (18 h) and 90% (48 h). Consistent with PSA promoter activity, PSA protein level also was decreased by ≥ 80% in cells treated with BIRM (Figure 1B, Insert). Since AR signaling (PSA promoter activity) was blocked by BIRM treatment, we next explored the effect of BIRM on AR protein expression levels in AR expressing cells. We found a reduction of AR protein expression in both LNCaP and LAPC-4 cells from as early as 3 h following BIRM treatment (Figure 1C) and further, dose dependent AR decrease was also observed in LAPC-4 with concomitant decrease in the phosphorylated-AR (Figure 1C). Furthermore, we examined whether BIRM affects AR translocation to the nucleus. LAPC-4 cells were treated with 5 μl/ml BIRM for 24 h and AR levels in cytosol and nucleus were determined by first isolating the two fractions as described in methods or using a kit (NE-PER Nuclear and Cytoplasmic extraction reagents, Thermo-Scientific, Cat#78833) then by western blotting of respective fractions. We found a significant decrease in AR levels in both cytosol and nucleus as found in whole cell extract, suggesting that BIRM did not affect AR translocation, but reduced the AR protein levels (Figure 1D).

### BIRM accelerates AR degradation by ubiquitin/proteasome pathway

**Figure 2:**
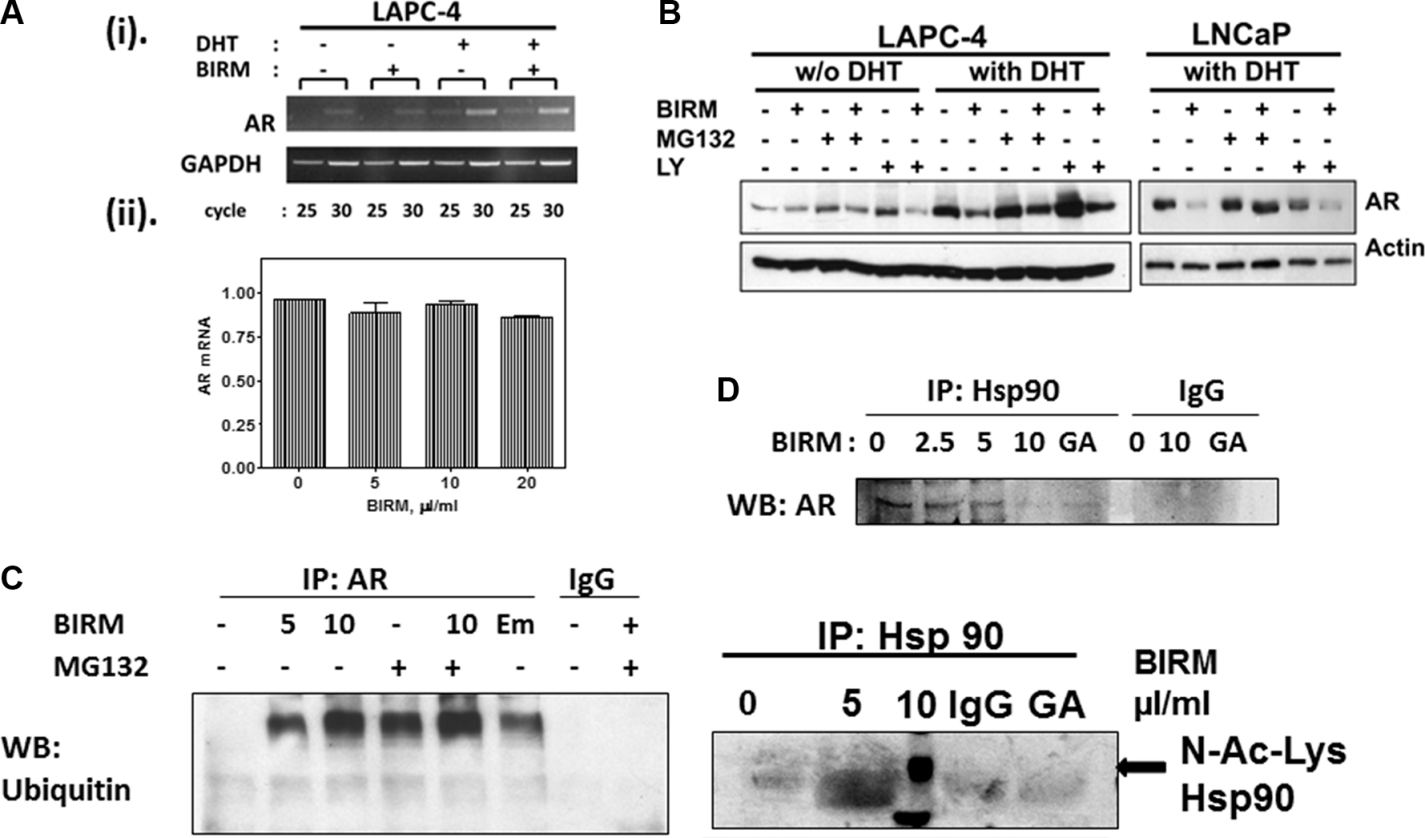
BIRM induced decrease in AR is due to increased proteasomal degradation of AR (**A**) Total RNA was purified from LAPC-4 treated with 10 μl/ml BIRM with or without 1 nM DHT for 48 h and semi-quantitative RT-PCR (i) was performed. GAPDH was amplified as an internal control. (ii) Quantitative real time PCR was performed on LAPC-4 cells treated with BIRM with 1nM DHT, the graph shows a relative AR mRNA expression normalized against a β-actin mRNA. (**B**) LAPC-4 and LNCaP cells incubated with or without BIRM (20 μl/ml) for 48 h, were pretreated with 10 μM LY294002 (PI-3Kinase inhibitor) for 1 h, or co-treatment with MG132 (proteasome inhibitor) for 4 h at the end of treatment. Decrease AR levels were monitored by Immunoblotting. (**C**) LAPC-4 cells were treated with indicated doses of BIRM for 48 h with or without MG132 treatment as described above. Immunoprecipitation was performed using anti-AR antibody and Western blotting was performed with anti-Ubiquitin antibody. Emodin treatment was performed for positive control of AR ubiquitination. (**D**) LAPC-4 cells were treated with various doses of BIRM for 48 h or 5 μg ml geldanamycin (GA) for 18 h and harvested. Cell lysate was used for Immunoprecipitation with a-Hsp90 antibody, separated by SDS-PAGE and probed with a-AR or a-acetyl lysine antibody. GA treated samples was used as a positive control of decrease interaction between AR and Hsp90. Due to high background in control band (0 μl/ml BIRM) in lane1, accurate densitometry of band intensity was difficult. However, there was > 50% decrease in the Hsp90-bound AR at 5 μl/ml BIRM. BIRM induced strong acetylation of Hsp90 at 5μl/ml. The disintegrated band at higher BIRM exposure might be associated with cells undergoing apoptosis. GA: Geldanamycin treated cell lysates, IgG: normal mouse IgG as negative control in IP.

Since AR protein expression was diminished in BIRM-treated cells, we next investigated whether AR down-regulation was by inhibition of AR synthesis or degradation. The effect of BIRM on AR synthesis was determined by qPCR in LAPC-4 cells. BIRM did not affect the AR mRNA significantly as confirmed by semi-quantitative PCR [Figure 2A (i)], qPCR [Figure 2A (ii)] and AR promoter activity (data not shown). BIRM did not alter the AR mRNA levels even under androgen deprivation condition suggesting that *de novo* AR synthesis was not suppressed by BIRM treatment.

We next examined whether BIRM affects AR stability, specifically whether BIRM increases the rate of AR degradation. AR is degraded via ubiquitin/proteasome pathway activated by PI3K-Akt signaling pathway [19]. Therefore, LAPC-4 and LNCaP cells (with DHT) were treated with combination of BIRM, proteasome inhibitor MG132 and PI3K inhibitor LY294002. AR levels were determined by immunoblotting. As shown in Figure 2B, AR levels were low without DHT but were not affected by BIRM, or MG132. They were stabilized in cells treated with LY294002. However, BIRM treatment decreased the level of AR protein with or without LY294002 suggesting, AR stability in dormant state is independent of phosphorylation by AKT. However, in the presence of DHT, AR levels in BIRM treated cells were significantly lower (≥ 50%) than that in untreated cells, and further, co-treatment with S26 proteasome inhibitor, MG132 prevented this depletion. This rescue by MG132 was similar in both cell lines expressing AR (LNCaP and LAPC-4). We also observed similar rescue by another proteasome inhibitor, epoxomicin (an inhibitor of 22S proteasome; data not shown).

To further substantiate BIRM-mediated accelerated AR degradation is through ubiquitination, we performed co-immunoprecipitation of AR and ubiquitinated AR from LAPC-4 cells with or without pre-incubation with BIRM. We found BIRM mediated ubiquitination was dose dependent (Figure 2C), comparable to protein treated with emodin (Figure 2C), a drug that was shown previously, to increase AR-ubiquitination and accelerated degradation [33–34]. Furthermore, co-treatment with BIRM and MG132, an inhibitor of S26 proteasome inhibitor that slows degradation of ubiquitinated-AR, showed abolition of AR-degradation, indicating ubiquitination and subsequent degradation of AR, is the major cause of AR down-regulation by BIRM.

### BIRM destabilizes HSP90-AR complex

Ligand-free AR, along with other co-factors, forms a heteromeric complex with Hsp90 that stabilizes ligand-free AR [20–25]. Previous studies have shown that a kinase inhibitor (i.e. Emodin) and Hsp90 inhibitor (i.e., Geldanamycin) disrupt the association between AR and Hsp90 [24, 25]. Therefore, we next examined the effect of BIRM on AR-Hsp90 formation. As shown in Figure 2D (i), BIRM exposure significantly disrupted the association between AR and Hsp90 in LAPC-4 cells. BIRM (5 μl/ml, 4 h) abolished AR-Hsp90 interaction by > 90% (Figure 2D, top panel), comparable to that by Geldanamycin (GA), an HSP90 inhibitor. Hyperacetylation of Hsp90 has been shown to inactivate Hsp90 and relieve bound protein off this chaperone [25, 26]. This prompted us to examine acetylation levels of Hsp90 after BIRM treatment. We found that treating cells with BIRM caused hyper acetylation of Hsp90 [Figure 2D (ii)] as shown by probing the western blot with anti-N-acetyl lysine antibody. This observation indicated that BIRM caused hyper acetylation and inactivation of Hsp90, resulted in disruption of AR-Hsp90 interaction, and further degradation of AR.

### BIRM disrupts cell survival pathway and stimulates apoptosis

**Figure 3:**
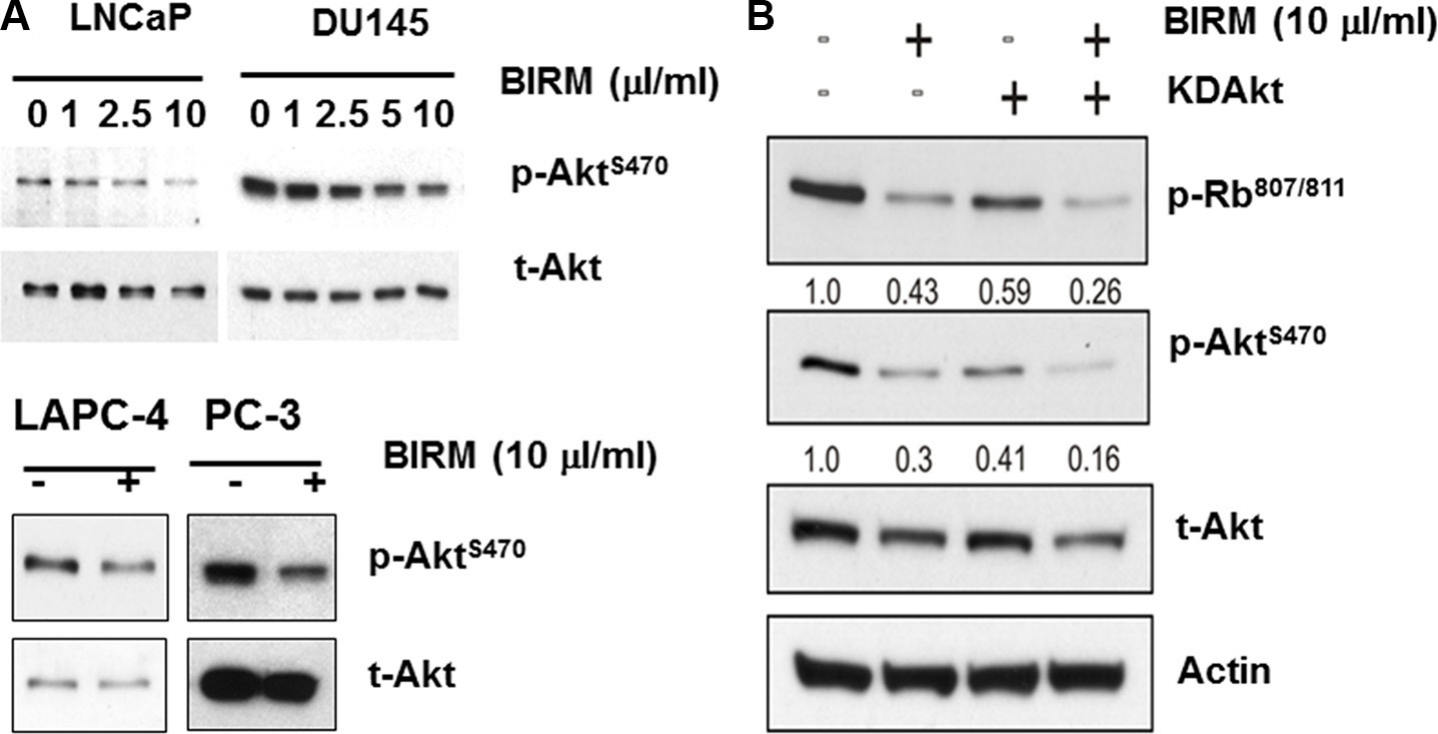
Effect of BIRM in phosphorylation of Akt (**A**) LNCaP, LAPC-4, PC-3 and DU145 cells were treated with various doses of BIRM for 48 h and p-Akt levels were monitored by Immunoblotting. Relative levels of p-Akt against t-Akt were calculated compared to untreated samples. (**B**) LAPC-4 cells were transiently transfected with either KDAkt or P^PCDNA3^ (control). Then, cells were treated with 10 μl/ml BIRM for 24 h. KDAkt: Kinase-Dead domain containing AKT transfectants.

Since AKT regulates several survival pathways in normal and tumor cells, we examined whether BIRM treatment alters AKT activity in PCa cells. Since AKT mediated survival pathway is common among all PCa cell lines, we examined the levels of phosphorylated AKT (pAKT) in both androgen-responsive and castration resistant PCa cell lines (LNCaP, LAPC-4, DU 145 and PC-3). As shown in Figure 3A, BIRM treatment decreased p-Akt^ser473^ levels in a dose-dependent manner. We hypothesized that hypo-phosphorylation of Akt by BIRM contributes G1 cell cycle arrest. To elucidate that, we transiently transfected the LAPC-4 cells with a plasmid coding for kinase-dead Akt (KD-Akt) [27]. Transfected cells were incubated with BIRM for 24 h and p-Rb levels were monitored. As expected, BIRM treatment caused dephosphorylation of Rb protein (Figure 3B). Transient expression of KD-Akt decreased p-Rb levels, compared to that of control (Figure 3B). Furthermore, combination of BIRM and KD-Akt caused further decrease in p-Rb levels, suggesting that dephosphorylation of Akt by BIRM treatment is correlated with BIRM mediated cell cycle arrest at G1 [18]. We used SH5, a specific inhibitor of Akt, to further corroborate our observation that BIRM induced cell cycle blockade at G1 is mediated by inhibition of AKT activation [27, 28]. We obtained same results with or without BIRM treatment as with KD-Akt which, further confers that Akt involvement is correlated with BIRM mediated cell cycle arrest at G1 phase.

### BIRM induced apoptosis is due to the activation of death-inducing signaling complex (DISC) or extrinsic pathway

**Figure 4:**
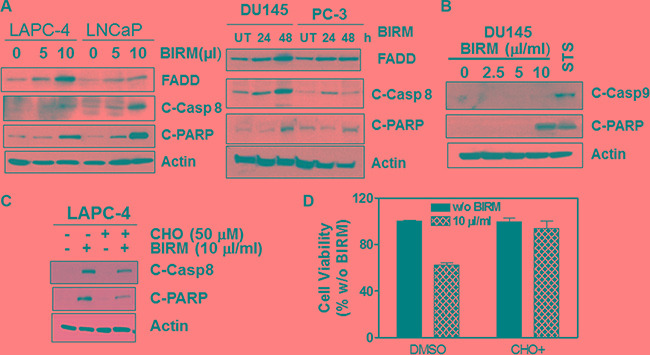
BIRM induces apoptosis by extrinsic (DISC) pathway (**A**) FADD, Cleaved caspase-8 and cleaved PARP levels were monitored in LAPC-4 and LNCaP cells treated with indicated doses of BIRM for 48 h (left panel), and DU145 and PC-3 cells treated with 10 μl/ml BIRM for indicated time points (right panel) by Immunoblotting. (**B**) DU145 cells were treated with BIRM for 48 h or staurosporine (STS) for 18 h and indicated protein levels were monitored by Immunoblotting. (**C**) LAPC-4 cells were pretreated with 50 μM caspase-8 inhibitor (Ac-IETD-CHO) for 1 h, followed by 50 μl/ml BIRM for 48 h. Then, cells were harvested for Immunoblotting to detect cleaved caspase-8 and PARP. (**D**) DU145 cells were pretreated with 1 μM CHO for 1 h, followed by BIRM for 48 h. Cytotoxicity of BIRM was monitored by MTT assay. The data presented as means ± SEM from two or more independent experiments in triplicate. FADD: Fas-Associated via Death Domain. PARP: Poly-ADP-Ribose Polymerase; C-PARP: Cleaved PARP; UT: Untreated; C-Casp 8: Cleaved (activated) caspase 8; C-casp9: Cleaved (activated) caspase 9; STS: Staurosporin. CHO: Inhibitor of Caspase 8 activation.

We reported previously that BIRM caused executioner caspase (e.g., caspase-3) activation in all PCa cells [18]. To further elucidate whether apoptosis induction by BIRM is mediated through extrinsic (death receptor mediated caspase-8 activation) or intrinsic (mitochondria depolarization) pathway, we measured the critical cellular determinants of extrinsic (caspase-8 activation) versus intrinsic (permeabilization of mitochondria) apoptosis pathways activated by BIRM. In all cell lines, FADD and active caspase-8 (cleaved caspase-8) were increased after BIRM treatment, resulted in increased level of cleaved PARP (Figure 4A). We also attempted to detect potential activation of Caspase 9, an initiator caspase that is activated by cytochrome-c release and formation of apoptosome via adapter protein AFAF-1 [29]. However, BIRM did not affect the caspase 9 activity in DU145 cells (Figure 4B) that implied, BIRM mediated apoptosis in PCa cells is unlikely due to “intrinsic” mechanism. Furthermore, LAPC-4 cells treated with BIRM resulted in significant increase in active caspase-8 (Figure 4C) that in turn activates caspase-3 (data not shown) and increased PARP cleavage. Moreover, induction of caspase-8 activation by BIRM was confirmed, by the absence of apoptosis when a specific inhibitor of caspase-8, (Ac-IETD-CHO) was co-incubated with BIRM. This inhibitor also suppressed BIRM induced increase in downstream apoptotic event, cleaved PARP (Figure 4C). The cytotoxic effect of BIRM on LAPC-4 cells was suppressed (31%) by Caspase-8 inhibitor when compared to vehicle control (Figure 4D).

### Antitumor activity of BIRM on PC-3ML cells

**Figure 5:**
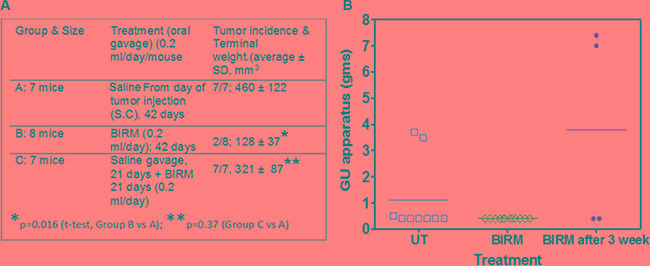
BIRM reduced incidence of PC-3 and LNCaP tumors (**A**) Summary of PC-3ML tumor growth in athymic mice. *Only two mice in BIRM treated group from day of tumor cell injection were palpable out of 8 mice. Comparison was made on average tumor weight of two mice vs, that from untreated mice (7 animals). (**B**) Tumor generated by orthotopic injection of LNCaP-EGFP cells. Three groups of athymic mice were injected with LNCaP-EGFP cells intraprostatically to dorsolateral prostate. Tumor incidence (9/30) control, and BIRM treated by providing in drinking water from second day of tumor implant (group 2: 12/15) and Group 3: BIRM was given 3 weeks after tumor implant (4/15) was noted in each of the three groups. No significant fluorescence was detected in GU organs of Group 2. Fluorescence was not uniform in any of the groups. Animals with tumors were euthanized after 95 to 124 days. The results presented are from one experiment. A significant number of animals died in all groups or were euthanized due to loss of body weight (not shown). As shown in the figure, at the time of necropsy, genitourinary system except the seminal vesicles was dissected and weighed to estimate the total weight of the GU apparatus. Error bars represent ± SD, *n* = 4 to 12.

Gavaging mice with BIRM for 42-days had no detectable effect on body weight or other forms of toxicity. As shown in Figure 5A Table, tumors were evident in 21 days where 21 of the 24 mice injected with PC-3ML tumor cells had palpable tumors. Mice gavaged with BIRM on the same day as of tumor cell injection showed significant decrease in tumor growth and terminal tumor weight (Group B) as compared to the mice in Group A where they were gavaged with saline alone (*p* = 0.016, *t-test*). Further, more significantly, only 2 out of 8 mice in Group B developed tumors beyond palpability. This result suggest BIRM has strong antitumor activity. However, in mice allowed to develop palpable tumor (14/16 mice) by 21 days after injection, gavage with BIRM from day 21 to 42 showed some small difference in tumor weight (321 ± 87 mm^3^ vs 460 ± 122 mm^3,^ *p* = 0.37) but not significant, suggesting BIRM is unlikely to have significant effect on established tumor in this castration resistant tumor model.

### Effect of BIRM on LNCaP tumors generated by orthotopic injection

We observed a low incidence of LNCaP tumors in the prostate (dorsal, ventral or lateral) gland of athymic mice. The incidence was about 36% in control (3/11; given normal drinking water) and 0% (0/11) in Group 2, where the animals were given 2% BIRM in drinking water continuously from the day tumor cells were injected. In the group of mice given BIRM, 3 weeks after tumor cell injection, the tumor incidence was 40% (2/5), similar to that observed in untreated control (Figure 5B).

### Preliminary purification of BIRM

**Figure 6:**
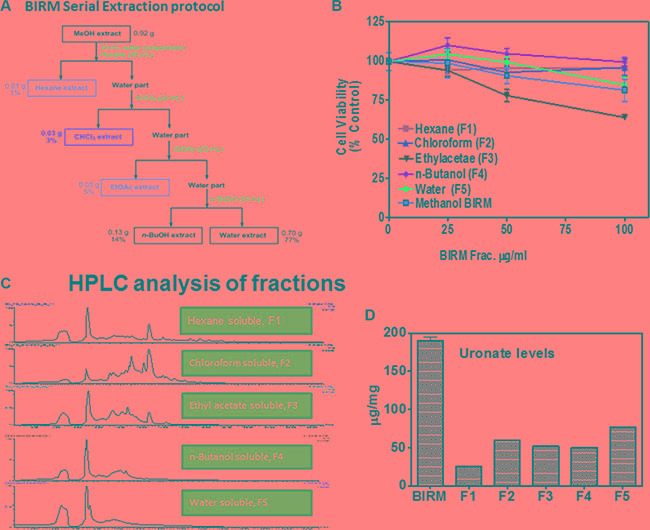
Fractionation of BIRM (**A**) Schematic representation of solid-liquid extraction of BIRM using different organic solvents and their yield. (**B**) Cytotoxicity BIRM fractions (F1-F5) extracted from different solvent in DU145 cells. (**C**) LC/MS chromatogram of all the fractions from F1-F5. (**D**) Uronic acid estimation in Fractions, note all fractions contained uronic acid containing GAG-like compounds.

About 16% (Vol/Vol) of BIRM, as extracted from ground roots, is insoluble fibrous matter. After centrifugation, the liquid BIRM with soluble fiber had a dry mass of ~ 40 mg/mL equivalent. For fractionation of active principle, BIRM was first extracted with methanol where most of the solid matter composed of aqueous-precipitable and methanol-soluble material (70%) were precipitated. The methanol soluble extract was evaporated using a Rota-evaporator, dried sample was reconstituted with water and extracted sequentially with different solvents: hexane, chloroform, ethyl acetate (EA) and n-butanol [29] by liquid-liquid extraction method. A complete description of the extraction process is summarized in the flow chart (Figure 6A). The yield of BIRM from various extracts were: 1% (F1), 3% (F2) 5% (F3) and 14% (F4) from hexane, chloroform, ethyl acetate and n-butanol, respectively. The highest yield (% of total weight) acquired from BIRM liquid-liquid extraction method was the water extract 77% (F5). The cytotoxic activities of these solvent extracts were tested on DU145 cells by MTT reduction assay. The fractions F1, F2 and F3, except F4 and F5 showed cytotoxicity on DU145 cells at ≥ 500 μg/ml. However the EA fraction showed 4-fold increase in specific activity (IC_50_ = 120 μg/ml) compared to BIRM (Figure 6B).

**Figure 7:**
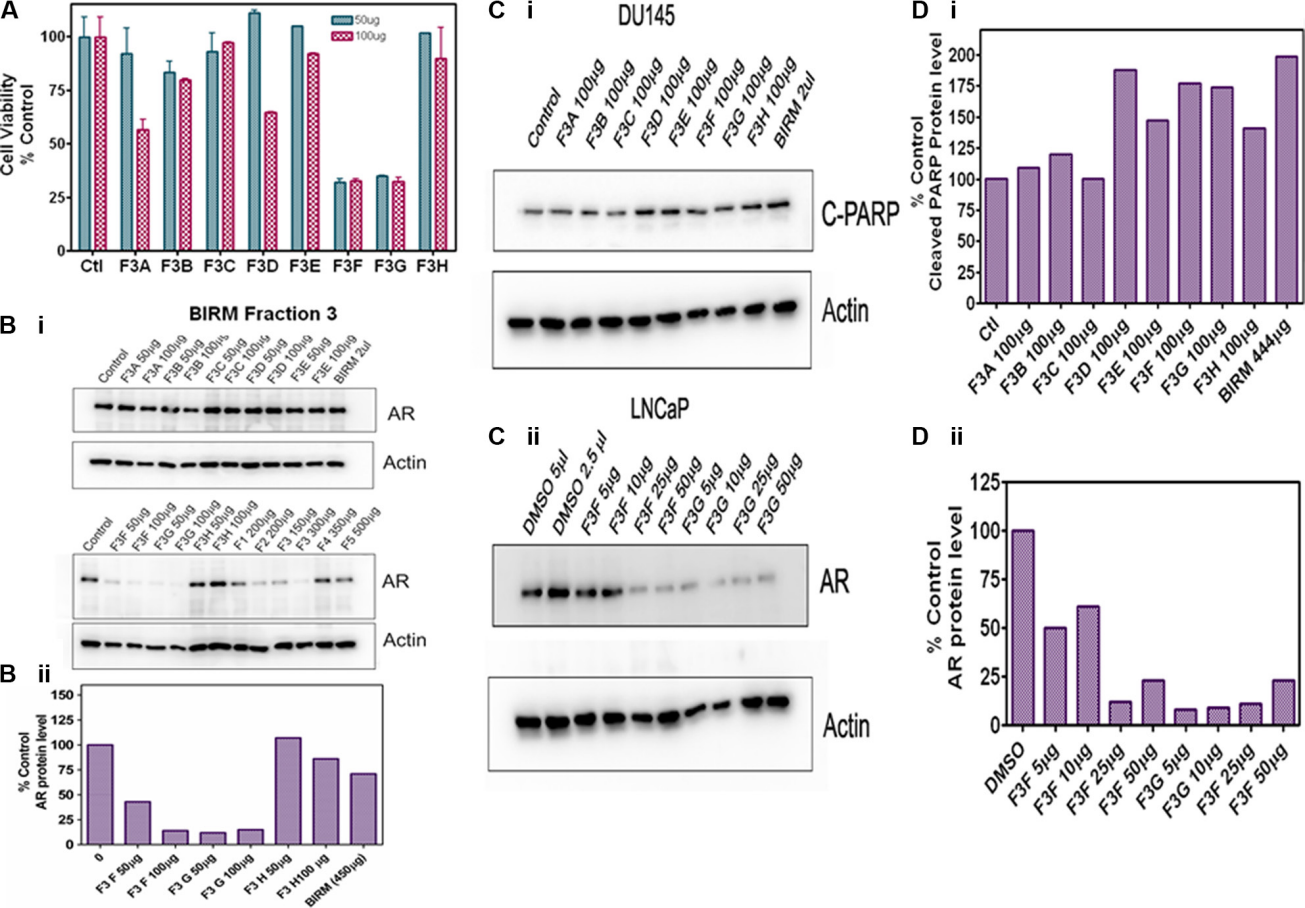
Biological activities of F3 sub-fractions Column fractions of F3 were assayed for both for cytotoxic activity and anti-AR activities. (**A**) Cytotoxicity of F3A-F3H on DU 145 cells at 50 and 100 μg/ml. (**B**) Anti-AR activities of BIRM fraction F3A-H. Several subfractions (F3F and F3G, and F2 and F3 had strong anti-AR activity when LNCaP cells were exposed to these fractions for 24 h. (Bii): densitometry trace of AR levels. (**C**) Apoptotic activities of BIRM sub-fractions. DU 145 cell cultures were exposed to F3 sub fractions and levels of cleaved-PARP was detected in cell lysates by Western blot. Only F3F and F3G had both pro-apoptotic and anti-AR activities. (**D**) Densitometry trace (estimate) of C-PARP (D.i) and AR (D.ii) are shown.

The fractions extracted from the solvents were subjected to LC-Mass spectrometry. The chromatogram profile showed a common peak in hexane, chloroform and EA fractions (Figure 6C). Further spectroscopic characterization of the common peak by proton NMR analyses showed a compound as benzoic acid, however it showed no specific activity in PCa cells and did not inhibit AR levels when tested on LNCaP cells (data not shown). Therefore, we reasoned, benzoic acid is unlikely to be an active anti-tumor agent in BIRM. Moreover, the LC-MS chromatogram profile of BIRM EA (F3) fraction showed multiple maxima other than benzoic acid. A sub-fractionation of EA by HPLC resulted in 8 peaks (F3A-F3H). The MTT cytotoxicity assay on these peaks showed an enhanced specific activity on F3F and F3G peaks compared to BIRM (Figure 7A). Furthermore the AR protein level was also reduced on peaks F3F and F3G [Figure 7B (i & ii)]. The induction of apoptosis by the peaks F3F and F3G on DU145 cells was more than 75% [Figure 7C (Ii & ii) and Figure 7D (i & ii)]. These results suggest that there are chemically closely related multiple active compounds in BIRM which attributes to its antiproliferative and anti-AR activities.

All three peaks that showed anti-proliferative activity against PCa cells were assayed for uronic acid content for identifying chemical nature of the MS-LC peaks. All the fractions had uronic acid content that varied from 25 ug to 80 ug. Further, we also analyzed these peak fractions for potential to inhibit HYAL1, a tumor specific hyaluronidase that was inhibited by BIRM [18]. These results suggested, the cytotoxic activity in BIRM is very likely correlated with the presence of HA-like compounds.

## DISCUSSION

These studies were conducted to demonstrate mechanistic basis of anti-tumor activity of BIRM specific to PCa. Our previous reports [18] presented the evidence of anti-neoplastic activity of BIRM on PCa cells. The activities of BIRM included inhibition of cell proliferation (arrested at G_0_/G_1_ in PC-3ML and LNCaP), inhibition of clonogenic growth of both androgen dependent (LNCaP) and castration–resistant PCa cells (DU145 and PC-3) and induction of apoptosis. More importantly, BIRM reduced incidence, delayed tumor growth and caused a significant decrease of metastasis of Dunning EGFP-MAT Lylu tumors (18). However the molecular target of BIRM and its mechanism of therapeutic action on prostate cancer was not investigated at that time.

Our present study showed the mechanistic action of BIRM inhibition of cell growth in both androgen dependent and independent PCa cells. LAPC-4 cells were more sensitive to BIRM mediated cell growth inhibition in the presence of DHT-mediated growth when compared to that in androgen deprived LAPC-4 cells (Figure 1A). BIRM caused an inhibition of PSA production in LAPC-4 cells with DHT (Figure 1B), suggesting that BIRM suppressed AR signaling even at the basal signaling level. A repressed AR signaling in androgen dependent PCa cells was due to BIRM mediated AR degradation (Figure 1C) via the S-26 proteasome mediated pathway (Figure 2B). Furthermore, treatment with BIRM caused neither inhibition of AR synthesis (Figure 2A) nor AR translocation (Figure 1D).

The inhibition of Hsp90 function results in degradation of targeted proteins, such as hormone receptors and cell cycle related protein. Hsp90 has been also reported that it is involved in AR folding for its maturation and stabilization [26]. As similar to Hsp90 inhibitor, geldanamycin, BIRM caused disruption of Hsp90-AR complex formation, and resulted in reduced level of AR due to its degradation (Figure 2Di). Furthermore, BIRM caused hyper acetylation of Hsp90 in PCa cells (Figure 2Dii), suggesting that BIRM mediated acetylation of Hsp90 may cause inhibition of Hsp90 function, which is consistent with previous report [22, 24, 25], resulted in AR degradation. This result gives us the hypothesis that BIRM may down-regulate not only AR, but also other Hsp90 targeted protein, such as ER and EGFR, via inhibition of Hsp90 functions, which are not investigated for this report.

We observed that BIRM reduced the levels of phosphorylation of Akt at ser 473 (Figure 4A) which is linked to survival pathway through activation of transcription of anti-apoptotic genes [27]. Interestingly, Zhang et al. [28] have reported that p-Akt phosphorylates and activates the winged helix family of transcription factors, FKHRL1, FKHR and AFX. This resulted in retaining FKHRL1 and FKHR in the cytoplasm. Further, inhibitors of mTOR, a downstream effector of the phosphatidylinositol 3-kinase (PI3K)/Akt signaling pathway, prevent cyclin dependent kinase (CDK) activation, inhibit Rb protein phosphorylation (at pRb^s780^), and accelerate the turnover of cyclin D1, leading to G1-phase arrest [28]. Moreover, it has been shown that FOXO fork head transcription factors down regulate cyclin D1 in a p27^kip1^-independent mechanism [27, 30]. Thus, we conjecture that decreased phosphorylation of Akt levels by BIRM results in G1 cell cycle arrest via downregulation of cyclin D1 and concomitant de-phosphorylation of Rb. In addition to acceleration of AR degradation, BIRM also showed cytotoxicity in both AR dependent and independent PCa cells. Apoptosis is tightly regulated by prosurvival and proapoptotic molecules, including proteins of the caspase family, and can be mediated by several different pathways. It is imperative to elucidate the mechanisms by which potential chemotherapeutic/chemopreventive drugs cause apoptosis. We showed that BIRM caused apoptosis in both AR positive and AR negative cell lines [18]. BIRM caused increase FADD, cleaved caspase-8 and PARP in all PCa cell lines examined (Figure 4A). Furthermore, BIRM caused cleaved caspase 8 but not caspase 9 (Figure 4B) The specific caspase inhibitor (Ac-IETD-CHO) reverted the action of BIRM induced apoptosis on LAPC-4 cells (Figure 4C) and a synthetic inhibitor of caspase-8 (Ac-IETD-CHO) rescued the BIRM induced cytotoxicity (Figure 4D) suggesting that BIRM induced apoptosis is through caspase-8 activated death-inducing signaling complex (DISC) pathway [31].

Previously, we showed that BIRM contains at least three molecular species of different mass that have cytotoxicity [18]. In this investigation, we further characterize the BIRM. The active compounds in BIRM are rich in uronic acid sugar containing poly- and oligosaccharides (Figure 6). Our data showed that BIRM contains several compounds, chondroitin sulfate-like, heparan-sulfate-like or hyaluronic acid oligosaccharide containing 10 sugar residues with some modification. Further investigations to identify the exact molecule contained in BIRM and to explore the mechanism of each component are underway.

These results suggest potential chemopreventive function of BIRM in both androgen-independent and androgen-sensitive PCa xenografts. These observation showed that BIRM has substantial stronger antitumor activity when given before the tumors are established, as shown by the lack of tumor incidence (75%–80% reduction) and slower growth of incident tumors (71% decrease in tumor volume, Figure 5. We have shown previously in the Dunning MAT Ly Lu rat tumor model, BIRM also significantly inhibits tumor metastasis [18]. Therefore, BIRM may be a potential candidate as an effective chemopreventive agent, for preventing the growth of primary and metastatic tumors.

## MATERIALS AND METHODS

### BIRM

BIRM was a gift from EcuaBIRM Inc. (Quito, Ecuador). BIRM was prepared by extracting the powdered roots of a medicinal plant widely referred to as “Dulcamara” or “Life Root” in Ecuador. The botanical identification of Dulcamara was performed at the Catholic University of Quito. The reference specimen from the identified plant, *Kalanchoe gastonis-bonnieri* [formerly known as Bryophyllum gastonis-bonnieri (Rayam-Hamet & H Perrier)] is deposited and maintained at the QCA Herbarium, Pontific a Catholic University of Quito, Ecuador. BIRM was prepared by organoleptic method of the Government of Ecuador certified contractor, Renase CIA LTDA following authentication of the starting material by QCA Herbarium. The roots were dried at 65°^C^ in an indoor oven and the liquid extraction of BIRM was processed as per the EcuaBIRM established proprietary standards; each batch was certified by the Department of Sanitation, Government of Ecuador. Before use, BIRM was clarified by centrifugation at 10, 000 g for 10 min, supernatant that contained active principle and solubilized fiber were separated from insoluble fiber and used for all *in vitro* experiments. BIRM used in oral gavage to test its anti-tumor activity *in vivo* was not separated from insoluble fibers before. Further standardization of the product used in the present study is described under Results (in section: molecular characterization of BIRM).

### Cells and culture conditions

Cell lines LNCaP and DU-145 cells (ATCC, Manassas VA) and PC-3ML (a gift from Dr. M. E. Stearns, Drexel University, Philadelphia, PA) [32] were maintained in RPMI-1640 medium with 10% fetal bovine serum (FBS, Mediatech, Inc. Herndon, VA) and 10 mg/ml gentamicin (Invitrogen, Inc., NY). AR dependent LAPC-4 cells were obtained from Dr. Charles Sawyer's laboratory in UCLA (Los Angeles, CA) and cultured in Iscove's media (Invitrogen) containing 1nM DHT (Sigma, St. Louis, MO), 7.5% FBS and 10 mg /ml gentamicin. The cell lines used in the study were authenticated for their origin by Genetica DNA laboratories Inc. Cincinnati, OH).

### Other materials

LY294002 (PI3 kinase inhibitor), IETD CHO (Caspase-8 inhibitor), MG132 and epoxomicin (proteasome inhibitors) were purchased from Calbiochem (San Diego, CA). Geldanamycin was purchased from Stressgen (Victoria, BC, Canada). Staurosporin was purchased from Cell Signaling (Beverly, MA).

### RNA isolation and semi-quantitative RT-PCR and Q-PCR

Total RNA was isolated from untreated and BIRM treated LAPC-4 cells with RNeasy Mini kit (Qiagen. com). RNA was reverse transcribed and PCR-amplified, expressions of AR and PSA mRNA were analyzed by semi-quantitative RT-PCR and real time quantitative PCR (qPCR) using Bio-rad icycler IQ as described before [33, 34].

### Determination of antiproliferative activity of BIRM

We used both colorimetric (MTT) and [^3^H-Methyl] deoxy-thymidine [^3^H-TdR] incorporation assays to determine cell viability and DNA synthesis activity of PCa cell cultures treated with BIRM (0–20 ml/ml). The cytotoxicity of BIRM on PCa cell cultures was measured using Thiozoyl Blue (3-(4, 5-dimethylthiazol-2-y)-2, 5-diphenyltetrazolium bromide (MTT) reduction assay [33].

### Analysis of protein expression by Western blotting

Relative levels of various cellular proteins were determined by immunoblotting using a routine procedure described before [33]. After BIRM treatment, whole cell extracts were prepared in ice-cold NP-40 buffer (50 mM Tris pH8.0, 1% NP-40, 150 mM NaCl, 2 mM EGTA, 2 mM EDTA, 50 mM NaF, 0.1 mM NaVO4 and Protease inhibitor cocktail). Protein samples were dissolved in 2X loading buffer, separated by 8–16% gradient SDS-PAGE gel (LifeGel, Frenchs Forest, NSW, Australia) or 10% SDS-PAGE gel and transferred onto Immunobilon-P (Millipore, Bedford, MA). Indicated proteins were probed with specific antibodies. Antibodies to AR (Ab-1) and cyclin B1 (Ab-3) were purchased from LAB VISION (Fremont, CA). Anti-phospho-AR (Ser213/210) IgG was purchased from IMAGENEX (San Diego, CA). Anti-cleaved PARP (Asp214), FADD, phopho-FADD (Ser194), p-Akt (Ser 473), p-RB and cleaved caspase-8 (Asp374) were purchased from Cell signaling. Antibodies to β-actin (actin), and Akt were purchased from BD Pharmingen (San Diego, CA). Antibody to Hsp-90 was from Stressgen (Victoria, BC, Canada). The immunoblots were visualized by an enhanced chemiluminescence kit (ECL; Amersham, Piscataway, NJ). For Immunoprecipitation, cells were lysed in NP-40 buffer, mixed incubated with either anti-AR Antibody (LAB VISION) or anti-Hsp 90 IgG and incubated for 2 h in cold room with rotation. Then, Protein G Agarose Beads (Pierce, Rockford, IL) was added to the mixture and incubated for overnight at 4ºC. Immunocomplexes were washed with a washing buffer (0.3% FBS, 1% NP-40 in PBS) four times, and dissolved in 2 X SDS-sample buffer. SDS-Solubilized sample aliquots were fractionated on SDS-Polyacrylamide gel electrophoresis (SDS PAGE). Gels were blotted onto PCD-membrane and the resulting blot was sequentially incubated with blocking buffer (5% non-fat milk in PBS with 0.05% Tween 20) anti-polyubiquitin antibody (Zymed, South San Francisco, CA) to detect ubiquitinated AR or anti-AR antibody (LAB VISION) to examine the interaction between Hsp90 and AR. Co-Immunoprecipitation followed by western blotting was used to determine AR-HSP90 association with a technique as described before [33]. Band density was normalized to β-actin by re-probing the blot with anti-β actin antibody (Santa Cruz Technology Inc., Dallas, TX).

### Luciferase reporter assays

Activation of AR and PSA promoters [34, 35] were assayed in cells transfected with an AR or PSA promoter luciferase reporter constructs as described before [36, 37]. Briefly, LAPC-4 (1 × 10^4^) cells were co-transfected with PSA-luc along with pRL-TK Renilla using Effectene reagent (QIAGEN, Inc., Valencia, CA). Twenty four hours after transfection, cells were treated with indicated doses of BIRM for several time points before measuring the luciferase activity using the Duo Glo luciferase kit and the Glomax Plate reader (Promega Corporation, Madison, WI). Relative Light Units (activity, RLU) were normalized to TK-Renilla activity.

### Preparation of nuclear and cytosolic fractions

Cell fractionation was performed to detect AR levels in nuclear and cytoplasmic fractions. Briefly, the procedure involved: after treatment with BIRM, cells were trypsinized and washed with cold PBS once. Then, cells were lysed in ice-cold CSK buffer (10 mM PIPES pH 6.8, 100 mM NaCl, 300 mM sucrose, 3 mM MgCl2, 1 mM EGTA, 0.5% Triton X-100 with protease inhibitor cocktail) for 10 min on ice. The cytosolic fractions as supernatant was collected by centrifugation. The nuclear fraction as pellet was washed once with CSK buffer, resuspended in buffer C (20 mM HEPES, pH 7.9, 0.4 M NaCl, 1 mM EDTA, 1 mM EGTA, 1 mM DTT and protease inhibitor cocktail) and kept on ice for 30 min, then, collected by centrifugation. Both fractions were boiled in 2X SDS-Gel sample buffer for 3 min and stored at 4ºC awaiting anlysis by Western blotting. Cytoplasmic AR was normalized against β-actin and nuclear AR was normalized using PARP antibody respectively.

### Tumor generation and treatment

(i): We used FOXN1 inbred athymic male mice were purchased from Charles River Laboratories (Durham, NC) for all studies reported here. A power analysis was performed for determining sample size for each of the experiments. The power analysis showed that for PC-3ML tumors, with take rate of > 90%, we needed 7–8 animals per group to get 80% confidence [38]. We injected 6-week old mice with 2 × 10 ^6^ cells/site/per animal in 50% Matrigel on the dorsal flank. Mice were treated with BIRM by oral gavage (0.2 ml/mouse/day; for up to 21 days) as described in Figure 5A. Tumor growth over time was evaluated by tumor volume measurement followed by tumor weight estimation following euthanasia on day 42. (ii): Orthotopic implantation and treatment: We injected LNCaP cells over expressing Enhanced Green Fluorescent Protein (EGFP-LNCaP) in the dorsolateral prostate of FOX N1 nude mice as described before using the procedure adapted from Stephenson et al [39]. Briefly, we performed orthotopic implant of EGFP-LNCaP cells on 10 mice for each control and experimental groups. Mice aged 6 to 8 weeks were anesthetized using isoflurane in a ventilated facility. Anesthetized mice were placed on the stage of a dissection microscope in the sterile hood to perform survival surgery. Abdomen was opened with a midline incision to expose prostate and ~ 0.02 ml suspension of 2 × 10^6^ EGFP-LNCaP cells was injected into the posterior prostatic lobe with a Hamilton push-button syringe fitted with a # 30 needle. A distinct blob of cells in the prostate indicated proper injection. Animals were sutured in two layers with internal chromic gut sutures and outside with silk sutures. Animals were allowed to recover under warming light until they regained movement after which they were injected with 0.01 ml of Buprenorphine every 12 hours for three days. Animals were examined under a Fluorescence *in vivo* imaging devise (Light Tools Research, Encinitas, CA) every two weeks, until the animals were euthanized at the end of 12 weeks. Although tumors could not be palpated reliably with fingers, fluorescence signal from EGFP-LNCaP cells was detectable percutaneously in 6 weeks and beyond.

### Treatment with BIRM

Group 1, the control group, received normal autoclaved drinking water given to athymic mice. Animals in Group 2 were given 0.2% BIRM in drinking water from the day of tumor cell injection, and Group 3 were given 2.0% BIRM starting 3 weeks after tumor cell injection. The BIRM containing water was replaced two times a week.

### Detection of tumors and estimation of antitumor efficacy

Tumor cell injected mice were anesthetized and imaged every two weeks using the *In vivo* imaging device [that captures fluorescence in a cooled-CCD video camera] and digital images were captured using the software provided by the devise vendor. All animals were euthanized at the end of 12 weeks, when two animals in control group had developed large tumors. At necropsy, the skin was removed to expose the tumors and fluorescence was imaged using the CCD camera. Following imaging, the entire genitourinary apparatus (bladder, and all prostate lobes), minus the seminal vesicle was removed and its weight was recorded. A part of the tumor was fixed and a histology was performed. Quantification of fluorescence image to estimate tumor size was not accurate due to poor distribution of fluorescent intensity along the curvature of the tumor, therefore are not presented. However, tumor weight from site of injection was quantified at the time of necropsy.

### Chemical characterization of BIRM

Three separate batches of BIRM, each manufactured by EcuaBIRM INC, were analyzed for chemical composition that included, elemental analysis, analysis of organic components, and macromolecular composition, MS and ^1^H and C^13^-NMR. These analyses were done at the Mikroanalytisches Labor Pascher, Remagen-Bandorf, Germany as reported previously [40]. Elemental analysis for relative abundance of C, H, and N, was determined by oxidative micro-combustion followed by micro-titration. Percent oxygen in BIRM was determined by the hot gas extraction followed by analysis on an infrared-analyzer. Sulfur and metal analyses were also performed using Inductively Coupled Plasma Atomic Emission Spectroscopy. Other chemicals, such as alkaloid by TLC [41], polyphenol using the Folin-Ciacalteu method [42] and tannins by ferric chloride protein precipitation assay [29] and uronic acid content in BIRM and its fractions were also conducted [43, 44].

## Abbreviations

AR: Androgen receptor
BIRM: Trademark name from EcuaBIRM Inc.
C-PARP: Cleaved (~85KD) fragment of Poly ADP-Ribose-Polymerase-1 (PARP)
DHT: Dihydrotestosterone
EGFP: Enhanced green fluorescent protein
GA: Geldanamycin
MTT: (3-(4, 5-dimethylthiazol-2-y)-2, 5-diphenyltetrazolium bromide
PCa: Prostate cancer
ECM: extracellular matrix
RT-PCR: Reverse transcriptase-PCR
Q-PCR: Quantitative RT-PCR
C-Casp9: Cleaved Caspase-3
STS: Staurosporine.
